# Telomeres and Thyroid Cancer

**DOI:** 10.2174/138920209789503897

**Published:** 2009-12

**Authors:** Marco Capezzone, Stefania Marchisotta, Silvia Cantara, Furio Pacini

**Affiliations:** Department of Internal Medicine, Endocrinology and Metabolism and Biochemistry, Section of Endocrinology and Metabolism, University of Siena, Siena, Italy

**Keywords:** Thyroid cancer, telomeres, hTERT.

## Abstract

Telomeres are specialized structures at the ends of chromosomes, consisting of hundreds of repeated hexanucleotides (TTAGGG)n. Genetic integrity is partly maintained by the architecture of telomeres and it is gradually lost as telomeres progressively shorten with each cell replication, due to incomplete lagging DNA strand synthesis and oxidative damage. Telomerase is a reverse transcriptase enzyme that counteracts telomere shortening by adding telomeric repeats to the G-rich strand. It is composed of a telomerase RNA component and a protein component, telomerase reverse transcriptase. In the absence of telomerase or when the activity of the enzyme is low compared to the replicative erosion, apoptosis is triggered. Patients who have inherited genetic defects in telomere maintenance seem to have an increased risk of developing familial benign diseases or malignant diseases. At the somatic level, telomerase is reactivated in the majority of human carcinomas, suggesting that telomerase reactivation is a critical step for cancerogenesis.

In sporadic thyroid carcinoma telomerase activity is detectable in nearly 50% of thyroid cancer tissues and some authors proposed that the detection of telomerase activity may be used for differentiating between benign and malignant thyroid tumours. Recently a germline alteration of telomere-telomerase complex has been identified in patients with familial papillary thyroid cancer, characterized by short telomeres and increased expression and activity of telomerase compared to patients with sporadic papillary thyroid cancer.

In this report, we will review the role of telomere-telomerase complex in sporadic and familial thyroid cancer.

## INTRODUCTION

Thyroid cancer (TC) is the most common endocrine malignancy and its incidence has increased significantly in many countries over the past three decades [1, 2]. TC comprises a group of tumors with different features and are subdivided into two major categories, depending on the cell type involved: 1) carcinomas originating from the follicular epithelium including the papillary (PTC), follicular (FTC) and hurthle (HTC) cell histotypes (Differentiated thyroid cancers, DTC) and the undifferentiated or anaplastic (ATC) histotype; 2) carcinomas originating from the parafollicular thyroid C cells, referred to as medullary thyroid cancer (MTC). Less frequent tumors include sarcomas, lymphomas, malignant hemangioendotheliomas, and metastases from other malignancies [3].

DTC are characterized by somatic mutations in the MAP-Kinase pathways represented by RET-PTC and TRK rearrangements, BRAF and RAS point mutations and PAX8-PPARγ rearrangements While BRAF, RET-PTC and TRK are limited to the papillary histotype, PAX8-PPARγ rearrangements are limited to the follicular histotype and RAS mutations are shared in both histotypes. In general these mutations are mutually exclusive and all together are found in nearly 80% of the cases [4]. 

DTC is prevalently sporadic, but evidence of a familial clustering is accumulating over the last years with prevalence up to 10% in different series [5, 6]. It is indicated as familial non-medullary thyroid carcinoma (FNMTC) and it is defined as the presence of thyroid cancer of follicular cell origin in two or more first-degree relatives [6]. The clinical presentation of these familial forms is heterogeneous with three different phenotypes: rare syndromes in which thyroid cancer is associated with non-thyroid disorders such as the Adenomatous polyposis of colon (FAP) [7], Cowden syndrome [8], Gardner syndrome [9], Werner syndrome [10] or Carney complex [11]. Other rare phenotypes are characterized by the association between FNMTC of particular hystotype with well defined susceptibility loci: familial PTC associated with papillary renal neoplasia (fPTC/PRN) linked to locus 1q21 [12], oxyphilic thyroid carcinoma (TCO) linked to locus 19p13 [13] and familial thyroid carcinoma follicular variant type 1 linked to locus 2q21 [14]. More frequently, FNMTC appears as the only manifestation in absence of any syndrome and no candidate genes. In this case, one study, where the author applied an exact probability measure to a series of first-degree family members suggested that the presence of only two affected members in kindred may represent a fortuitous association of the disease [15]. According to his mathematical simulation, 62-69% of 2-hit families are sporadic occurrences and thus, only families with ≥ 3 affected first-degree relatives should be considered for clinical and genetic investigations of FNMTC.

Telomeres are non-coding regions at the end of eukaryotic chromosomes consisting of hundreds of copies of a simple tandem repeat sequence (TTAGGG in vertebrates) that serves to stabilize the chromosome for replication through cell division. Telomeres are maintained by telomerase, a specialized ribonucleoprotein complex that includes an RNA template (*TERC*) and a reverse transcriptase catalytic subunit (*TERT*) [16]. Telomerase expression is low or absent in most of human somatic tissues, while it is expressed in germ and stem cell compartments. Telomeric DNA is dynamic, being progressively lost with each cell division due to incomplete replication of the ends of linear DNA. When telomeres become critically short, the cells undergo senescence or apoptosis but if the integrity of checkpoints mechanisms are altered genomic instability is triggered and leads to cycles of chromosome breakage and fusion, in a period called “crisis” that permit the acquisition of further genetic alterations [16]. Although most cells die by apoptosis during the “crisis”, rare cells survive and maintain stable short telomere lengths through the reactivation of telomerase that facilitates cell immortalization. This suggests that maintenance of telomere length is necessary for continued cell division and immortalization and both have been implicated in the control of the proliferate capacity of normal and malignant cells [17].

The strong association of telomerase re-activation with cancer provides evidence that this mechanism plays an important role in cancer development and telomerase activity (TA) can be regarded as a marker for human cancers [18]. Interestingly, patients who have inherited or acquired genetic defects in telomere maintenance seem to have an increased risk of developing familial benign diseases such as dyskeratosis congenital syndrome [19] and malignant diseases such as head, neck, lung, breast and renal cancers [20]. Some studies have shown that relative telomere length segregates in families [21, 22] and that decrease in telomere length may play a role in age-related genetic instability. High telomerase activity represents a mechanism of telomere stabilization which precludes DNA-damaged cells from apoptosis and contributes to their genomic instability and immortalization, or might represent an ineffective tentative of telomere repair. Coexpression of telomerase with oncogenes converts normal human cells into cells capable of tumorigenic growth [23] and these observations confirm that the acquisition of TA is not only a crucial step in attaining replicative immortality but also plays an important role in human cell transformation. Thus, alteration of the telomere-telomerase complex seems to have a role in development of both sporadic and familial cancer.

## TELOMERASE ACTIVITY IN NEOPLASTIC AND NON-NEOPLASTIC THYROID TISSUES

The analysis of TA is based on the telomeric repeat amplification assay (TRAP) [24]. In addition, it is possible to study the expression of hTERT through the RT-PCR detection of hTERT mRNA or immunohistochemistry with anti-hTERT monoclonal antibodies. 

In normal thyroid samples TA is almost absent. As showed in Table **1**, using TRAP assay, TA in normal thyroid specimens was found only in four studies, for a total of 12 cases out of 195 (6.1%) [25-28]. Few studies [26, 28-31] searched TA in benign thyroid disorders. TA was found in 4/27 cases of multinodular goiter (14.8%) and in 8/22 (36.3%) samples of autoimmune thyroid disorders. The higher incidence of TA in autoimmune disorders is not surprising since activated immune cells are known to have TA. Interestingly, Yashima [26] reported direct relationship between telomerase and degree of lymphocytic infiltration in normal, benign and malignant thyroid samples. Most of the studies analyzed benign (thyroid adenomas) and malignant nodules. In this setting large variations are present among different series and different methodologies. When using TRAP assay TA was found in 19.4% of 134 follicular adenomas from 11 series [25-38] and in 39.3% of 122 samples from 5 series [34, 35, 39-41] when using RT-PCR or immunohistochemistry. However, in those studies, telomerase activity/expression ranged 0-81% (Tables **1** and **2**). 

Among thyroid cancer increased TA or expression was found in all histotypes, (papillary, follicular, medullary and anaplastic), again with large variations in different series, but approximately approaching more than 50% of the samples (Tables **1** and **2**).

Umbrich *et al. *[32] in 1997 firstly reported the presence of TA in 100% of follicular thyroid carcinomas and the absence of TA in 76% of benign thyroid lesions. These authors stated that TA may provide a potential diagnostic marker distinguishing benign from malignant follicular thyroid tumors. Haugen *et al. *[25] reported the presence of TA in a large percentage of papillary thyroid carcinomas (PTC), but not in benign adenomas, follicular carcinomas, or most normal thyroid tissues. In contrast, Brousset *et al. *[33] reported that TA was present only in a minority of PTCs and was more frequently detected in follicular and undifferentiated carcinomas suggesting that telomerase may play some role in the pathogenesis of thyroid cancer known to have the most aggressive behaviour. Also Lo *et al. *[31] reported that TA does not appear to be frequently present in PTC. The authors hypothesized that the increased TA in PTC samples reported in other studies, may be due to the presence of lymphocytic infiltration coexisting with thyroid neoplasia.

Takano *et al. *[42] showed the presence of high levels of hTERT mRNA expression (by real-time PCR) in 12/12 ATC and six cell lines derived from an ATC, and in a considerable number of normal thyroid tissues (n=10), differentiated thyroid tumors (8 PTC, 6 FTC and 10 follicular adenomas) and medullary thyroid cancers (n=6). Interestingly, a recent paper [43] reported that the presence of downregulation of microRNA miR-138 expression may partially contribute to the gain of hTERT protein expression in ATC. 

Some studies [28, 36, 37, 44] showed that TA correlated significantly with the progression of the clinical stage suggesting that telomerase expression could be important in defining the clinical behaviour of thyroid carcinomas. One study [37] found TA in 42% of 50 PTC without lymphocytic infiltration and found that TA was more frequently present in advanced stages. Ito *et al. *[40] investigated hTERT expression by immunohistochemistry in 166 thyroid neoplasms. They reported progressive increase with advancing stage of neoplasm, from follicular adenomas (9.8%), FTC (39%) and PTC (34%) to ATC (73%). No hTERT expression was found in normal thyroid tissues. The author’s conclusion was that TA may contribute to anaplastic transformation of DTC. Only one study addressed the TA in young subjects (<21yrs) [44]. Of interest, tissue invasion, distant metastases and tumor recurrence develop exclusively in PTC that express telomerase, but not in telomerase- negative cases. In addition, comparing the PTC group with or without telomerase expression by immunohistochemistry, the authors found a shorter disease free-survival in telomerase-positive patients compared to telomerase-negative patients. Wang *et al*. [41] performed a case-control study to examine the expression of hTERT using immunohistochemistry in 36 FTC and 36 FA in age/sex matched patients and found that high hTERT immunostaining was significantly correlated with FTC, highlighting the important role of hTERT expression during the development of thyroid follicular cancer. Recently Wang *et al. *[45] reported in a group of 133 thyroid tumor tissues (60 malignant and 73 benign) a greater proportion of the active full-length hTERT transcript in malignant tumors, while in benign tumors they observed the presence of an inactive splice variant. Increased TA was limited to the expression of the full-length hTERT isoform. 

Foukakis *et al. *[46] evaluated by qRT-PCR 10 potential gene markers of malignancy in a panel of 75 follicular tumors that covered the entire clinical spectrum, from follicular adenomas, atypical follicular adenomas, minimally invasive FTCs up to metastatic and/or widely invasive FTC. Using logistic regression analysis, the authors were able to identify two genes, TERT and TTF3, whose combination was able to distinguish adenomas and low grade follicular carcinomas from aggressive follicular carcinomas. Their model correctly diagnosed as malignant some cases in which the clinical evidence of malignancy was based on the late development of metastases.

## TELOMERE LENGTH AND TELOMERASE ACTIVITY IN THYROID CANCER TISSUES

Telomere length is the result of a dynamic balance between elongation and shortening. Mean telomere length is species-specific and varies according to cell type [47]. Short telomeres have been associated to a higher risk of human carcinomas [48-50] including thyroid cancer [27, 29, 51, 52]. 

Kammori *et al. *[27] reported that TA is increased and telomere length (measured by Southern blot) is decreased in thyroid cancers and follicular adenomas compared to normal peritumoral tissues (Table **3**). Matthews *et al.* [29] examined TA and telomere length in a series of normal (n=10), benign (n=32) and malignant (n=53, 37 PTC and 16 FTC) thyroid samples, including an analysis of purified epithelial fractions to exclude lymphocyte contamination (Table **3**). Interestingly, on the basis of the correlation between TA and telomere length data, these authors suggested that thyroid cancers fall into three biological groups: telomerase-positive lesions, consistent with the conventional model of telomerase erosion followed by telomerase reactivation; telomerase-negative tumours, which maintain telomere length by a mechanism independent of telomerase; and telomerase-negative tumours which are still undergoing telomere erosion and may be composed of mortal cancer cells.

A study [51] in autonomous thyroid adenomas found shorter and variable telomere length compared to normal collateral quiescent tissue, with no telomerase activity to compensate the loss of telomere length (Table **3**). The absent reactivation of TA in autonomous adenomas does not allow the creation of a mechanism of cellular immortalization and explain why the telomeres of these cells, when have reached the short critical size, stop dividing. This is in agreement with the observations that many hyper-functioning adenomas encounter spontaneous necrosis and never become malignant.

Recently, Achille *et al. *[52] evaluated the expression of cell cycle regulatory proteins and telomere length in sporadic and radiation-induced PTC specimens and found that short telomere length did not differ significantly between sporadic and radiation-induced PTC. 

## TELOMERASE ACTIVITY IN FINE NEEDLE ASPIRATION CYTOLOGY (FNAC) OF THYROID NEOPLASMS

Fine needle aspiration cytology (FNAC) is the gold standard for the differential diagnosis between benign and malignant thyroid nodules. However the results of FNAC may be indeterminate and inconclusive in nearly 5-15% of follicular lesions. Numerous attempts have been made to clarify this “grey zone” of inconclusive diagnosis using potential markers of malignancy that might improve the diagnosis. The measurement of TA was proposed by several authors [32, 35, 39, 53-61] as a possible marker of malignancy that could help to increase FNAC diagnostic accuracy and thereby improve sensitivity in the preoperative management of thyroid nodules (Table **4**). 

Aogi *et al. *[53] reported a clear difference in detectable TA between FNAC samples that were histologically confirmed as malignant (83.3%) and those that were diagnosed as benign lesions (8.3%).

On the contrary, Sebesta *et al. *[54] reported that TA was detected in 64% of thyroid benign lesions and in only 60% of the malignant lesions concluding that the addition of telomerase assays failed to improve the sensitivity or specificity of thyroid FNAC.

Mora *et al. *[55] reported the results of TA in 102 thyroid nodules: 70 FNAC samples collected prospectively and 32 frozen tumors obtained retrospectively. In FNAC samples the presence of TA was detected in 44% of malignant nodules and in none of the benign FNAC. Among the 32 frozen tumors, TA was detected in 40% of TC but not in follicular adenomas.

Lerma *et al. *[56] measured TA in 147 FNAC consecutive samples of thyroid nodules and found that in the 120 samples without TA, cytology was indicative of benign nodules; on the contrary, TA was detected in 26% of the samples with cytologic diagnoses of thyroid cancer or follicular lesions. In conclusion, the detection of TA helped to confirm neoplasia (cancer or follicular adenomas) in 26% of suspicious thyroid nodules and although it was less sensitive than FNAC, TA specificity was 100% for any neoplasia and 87.5% for malignancy. 

Some authors [54, 58] considered that the detection of TA in cytological specimens does not add practical diagnostic information for the presence of false-positive results in patients with Hashimoto or lymphocitic thyroiditis. 

Guerra *et al. *[61] proposed that if a 10-Unit cut off as level of TA is established, detectable TA would be confined only to thyroid carcinomas and could be a useful marker in the diagnosis of thyroid cancer, especially in FNAC cases with indeterminate cytology.

Some authors reported that also RT-PCR for the hTERT gene may be useful as a preoperative method of evaluating thyroid lesions suspected of malignancy [35, 39, 41]. Zeiger *et al*. [39] examined hTERT gene expression by RT-PCR in 24 FNAC samples with suspicious or indeterminate cytology and found that nine of the 10 thyroid nodules benign at histology were negative for hTERT gene expression while 13 of 14 malignant neoplasms were positive for hTERT gene expression. 

Kammori *et al*. [57] examined TA in 6 FTC and 15 follicular adenomas and found the presence of TA in all FTC but only in 5 of the 15 (33%) follicular adenomas. Subsequently these authors evaluated hTERT gene expression by in situ hybridization (ISH) both in tissue sections and FNAC samples. In tissue sections they found the expression of hTERT mRNA in all FTC and in only one of the follicular adenomas; in FNAC samples they reported hTERT mRNA expression in 4 of the 6 follicular carcinomas and in 5 of the 15 adenomas. The authors concluded that hTERT mRNA expression in thyroid tumors might not be correlated with TA and that the detection of hTERT mRNA in FNAC samples using ISH cannot be used to definitively diagnose follicular tumors of the thyroid.

## RELATIVE TELOMERE LENGTH AND TELOMERASE ACTIVITY IN THE BLOOD OF PATIENTS WITH THYROID CANCER

The association of telomere length and hTERT activity in the blood of patients with thyroid cancers is less investigated. In 2004 Novakovic *et al*. [62] evaluated the significance of detectable expression of RNA for telomerase subunits hTR and hTERT in the plasma of 25 primary breast cancer patients, 29 patients with advanced malignant melanoma, 4 patients with advanced thyroid cancer (2 Hurthle cancers poorly differentiated, 1 papillary well differentiated and 1 anaplastic thyroid cancer) and 7 healthy volunteers. In healthy volunteers hTR was positive in 3 cases and hTERT was negative in all. In thyroid cancer patients, hTR and hTERT were positive in all. The authors stated that among the telomerase subunits, only hTERT could serve as an unspecific tumour marker in the plasma of cancer patients.

Recently the presence of an imbalance of the telomere-telomerase complex has been reported in the peripheral blood of familial papillary thyroid cancer (FPTC) patients [63]. Studying a series of 47 FPTC patients, 75 sporadic PTC, 20 patients with benign thyroid diseases, 19 healthy subjects and 20 unaffected siblings of FPTC patients, the authors observed that FPTC patients display shorter telomeres together with increased amplification in hTERT gene copy number and higher TA and expression compared to control groups. Telomere length (RTL) was measured by Q-PCR and FISH analysis. The mean of RTL by Q-PCR in FPTC patients was significantly shorter compared to sporadic PTCs and the other control groups. Relative telomere length (RTL) was also measured in 10 available cancer tissues of FPTC patients and compared with 30 sporadic PTCs. As in peripheral blood, the RTL was also significantly shorter in the tissues of FPTC patients respect to sporadic PTCs. The hTERT gene (measured by Q-PCR) was significantly amplified in FPTC patients compared to sporadic PTCs, healthy subjects, nodular goiter and unaffected siblings. Also TA was significantly higher in FNMTCs than in unaffected siblings and sporadic PTCs. 

The authors speculated that the high telomerase activity found in FPTC patients represents a mechanism of telomere stabilization which precludes DNA-damaged cells from apoptosis and contributes to their genomic instability and immortalization, or might represent an ineffective tentative of telomere shortening repair. These features (short telomeres and high telomerase activity) may be implicated in the inherited predisposition to develop FPTC. In addition, the observations reported in this paper correlate very well with another study from the same group [64] that demonstrated the presence of “genetic anticipation” (defined as the occurrence of a genetic disorder at progressively earlier ages and with increased severity in successive generations) [65], reinforcing the hypothesis that FPTC is a true familial disease rather than the fortuitous association of the same disease in a family.

## CONCLUSIONS

Reactivation of telomerase and presence of short telomeres have been found in a wide variety of human carcinomas. The presence of an imbalance in the telomere-telomerase complex has been also investigated in sporadic and in familial thyroid cancer. Regarding hTERT reactivation in thyroid cancer specimens, is not yet clear whether this represents just an epiphenomenon or, on the contrary, play a key role in the process of carcinogenesis. The different results reported in the literature may be due to different methodologies, and the role of different variables should be considered in the interpretations of the results. Therefore more prospective, case-controlled studies and standardization of the methods are needed.

The recent preliminary results reported at germline levels in FPTC, suggest that alterations in telomere/telomerase complex may be regarded as potential marker of familial cancer and could represent the first step to search for alterations in the genes regulating the length of telomeres. Furthermore, the same mechanisms reported in FPTC, might be implicated also in other forms of familial cancer (i.e. breast, colon, pancreas). 

A better definition of this issue can only derive from further studies in other series.

## Figures and Tables

**Table 1:** Meta-Analysis of Telomerase Activity (TA) in Neoplastic and Non-Neoplastic Thyroid Tissues

AUTHOR [Reference]	Normal pos/tot	FA pos/tot	PTC pos/tot	FTC pos/tot	MTC pos/tot	ATC pos/tot	Method
Haugen [25]	3/14 (21.4%)	0/4	10/14 (71.4%)	0/3	0/1	0/1	TRAP
Yashima [26]	3/22 (13.6%)	1/5 (20%)	4/11 (36.3%)	0/2	2/2 (100%)	-	TRAP
Kammori [27]	1/21 (4.7%)	3/9 (33.4%)	7/8 (87.5%)	3/3 (100%)	1/1 (100%)	-	TRAP
Onoda [28]	5/15 (33.4%)	0/4	9/16 (56.2%)	-	0/1	1/1 (100%)	TRAP
Matthews [29]	0/10	3/22 (13.6%)	8/37 (21.6%)	6/16 (37.5%)	-	-	TRAP
Aogi [30]	-	0/9	5/5 (100%)	3/3 (100%)	1/1 (100%)	1/2 (50%)	TRAP
Lo [31]	0/35	0/9	15/52 (28.8%)	0/2	0/2	1/2 (50%)	TRAP
Umbricht [32]	0/22	5/23 (21.7%)	-	11/11 (100%)	-	-	TRAP
Brousset [33]	0/20	1/12 (8.3%)	3/15 (20%)	4/6 (66.6%)	-	2/3 (66.7%)	TRAP
Hoang-Vu [34]	-	-	2/9 (22.2%)	6/9 (66.6%)	-	6/9 (66.6%)	TRAP
Saji [35]	0/10	-	20/30 (66.6%)	-	-	-	TRAP
Okayasu [36]	0/26	9/23 (39.1%)	16/26 (61.5%)	3/4 (75%)	-	2/2 (100%)	TRAP
Bornstein-Quevedo [37]	-	-	21/50 (42%)	-	-	-	TRAP
Cheng [38]	-	4/14 (28.5%)	12/23 (52.1%)	10/11 (90.9%)	-	-	TRAP
TOTAL pos/tot (%)	12/195 (6.1%)	26/134 (19.4%)	132/296 (44.5%)	46/70 (65.7%)	4/8 (50%)	13/20 (65%)	
RANGE (%)	0-33.4 %	0-39.1 %	20-87.5 %	0-100 %	0-100 %	0-100 %	

**Table 2:** Meta-Analysis of hTERT mRNA Expression in Neoplastic and Non-Neoplastic Thyroid Tissues

AUTHOR [Reference]	FA pos/tot	PTC pos/tot	FTC pos/tot	MTC pos/tot	ATC pos/tot	Method
Hoang-Vu [34]	-	3/9 (33.4%)	3/9 (33.4%)		6/9 (66.7%)	Immunohistochemistry
Saji [35]	2/7 (28.5%)	9/13 (69.2%)	6/6 (100%)	-	-	RT-PCR
Zeiger [39]	1/5 (20%)	8/8 (100%)	2/3 (66.7%)	-	-	RT-PCR
Ito [40]	4/41 (9.7%)	19/55 (34.5%)	20/51 (39.2%)	-	14/19 (73.7%)	Immunohistochemistry
Wang [41]	27/33 (81.8%)	49/52 (94.2%)	3/5 (60%)	-	-	RT-PCR
Wang [41]	14/36 (38.9%)	-	23/36 (63.9%)	-	-	Immunohistochemistry
TOTAL pos/tot (%)	48/122 (39.3%)	88/137 (64.2%)	57/110 (51.8%)		20/28 (71.4%)	
RANGE (%)	9.7-81.8%	33.4-100%	33.4-100%		66.7-73.7%	

**Table 3:** Meta-Analysis of Telomere Length in Neoplastic and Non-Neoplastic Thyroid Tissues

AUTHOR [Reference]	Normal (mean ± SD)	FA (mean ± SD)	Cancer tissue (mean ± SD)	Method
Kammori [27]	12.86 ± 2.76	10.38 ± 3.42	9.14 ± 3.15	Southern blot
Matthews [29]	7.2 ± 0.4	9.1 ± 0.8	7.4 ± 0.8 (FTC) 9.3 ± 0.5 (PTC)	Terminal Restriction Fragment
Deken [51]	12.3 ± 1.7	8.8 ± 1.6	-	Terminal Restriction Fragment

**Table 4:** Meta-Analysis of Telomerase Activity (TA) and hTERT mRNA Expression  in Thyroid Fine Needle Aspiration Cytology (FNAC)

AUTHOR [Reference]	Goiter	Adenoma	Total Carcinomas	Method
Umbrich [32]	2/24 (8.4%)	7/19 (36.8%)	42/47 (89.3%)	RT-PCR
Saji [35]	3/11 (27.3%)	2/7 (28.6%)	13/19 (68.4%)	RET-PCR
Zeiger [39]	0/5	1/5 (20%)	13/14 (93%)	RT-PCR
Aogi [53]	4/48 (8.4%)	-	6/8 (75%)	TRAP
Sebesta [54]	6/10 (60%)	3/4 (75%)	3/5 (60%)	TRAP
Mora [55]	0/57	0/1	4/9 (44.5%)	TRAP
Lerma [56]	0/5	1/3 (34%)	6/18 (33.4%)	TRAP
Kammori [57]	-	5/15 (34%)	6/6 (100%)	TRAP
Trullson [58]	0/15	0/15	6/16 (37.5%)	TRAP
Siddiqui [59]	2/7 (28.6%)	3/8 (37.5%)	16/24 (66.7%)	RT-PCR
Liou [60]	3/6 (50%)	3/6 (50%)	13/14 (92.8%)	RT-PCR
Guerra [61]	3/59 (5%)	0/6	20/20 (100%)	TRAP
TOTAL pos/tot (%)	23/247 (9.31%)	25/89 (28%)	148/200 (74%)	
RANGE (%)	0-60%	0-75%	33.4-100%	
